# The probable role of tissue plasminogen activator/neuroserpin axis in Alzheimer’s disease: a new perspective

**DOI:** 10.1007/s13760-023-02403-x

**Published:** 2023-11-02

**Authors:** Naif H. Ali, Hayder M. Al-kuraishy, Ali I. Al-Gareeb, Saud A. Alnaaim, Athanasios Alexiou, Marios Papadakis, Hebatallah M. Saad, Gaber El-Saber Batiha

**Affiliations:** 1 Department of Internal Medicine, Medical College, Najran University, Najran, Saudi Arabia; 2 Department of Clinical Pharmacology and Medicine, College of Medicine, ALmustansiriyia University, PO Box 14132, Baghdad, Iraq; 3 Clinical Neurosciences Department, College of Medicine, King Faisal University, Hofuf, Saudi Arabia; 4 Department of Science and Engineering, Novel Global Community Educational Foundation, Hebersham, NSW 2770 Australia; 5 AFNP Med, 1030 Vienna, Austria; 6 Department of Surgery II, University Hospital Witten-Herdecke, Heusnerstrasse 40, University of Witten-Herdecke, 42283 Wuppertal, Germany; 7 Department of Pathology, Faculty of Veterinary Medicine, Matrouh University, Matrouh, 51744 Matrouh Egypt; 8 Department of Pharmacology and Therapeutics, Faculty of Veterinary Medicine, Damanhour University, Damanhour, 22511 AlBeheira Egypt

**Keywords:** Alzheimer’s disease, Tissue plasminogen activators, Amyloid beta, Neuroserpin

## Abstract

Alzheimer’s disease (AD) is the most common type of dementia associated with amyloid beta (Aβ) deposition. Dysfunction of the neuronal clearance pathway promotes the accumulation of Aβ. The plasminogen-activating system (PAS) is controlled by various enzymes like tissue plasminogen activators (tPA). Neuronal tPA enhances the conversion of plasminogen to plasmin, which cleaves Aβ; this function is controlled by many inhibitors of PAS, including a plasminogen-activating inhibitor (PAI-1) and neuroserpin. Therefore, the objective of the present narrative review was to explore the potential role of tPA/neuroserpin in the pathogenesis of AD. PAI-1 activity is increased in AD, which is involved in accumulating Aβ. Progressive increase of Aβ level during AD neuropathology is correlated with the over-production of PAI-1 with subsequent reduction of plasmin and tPA activities. Reducing plasmin and tPA activities promote Aβ by reducing Aβ clearance. Neuroserpin plays a critical role in the pathogenesis of AD as it regulates the expression and accumulation of Aβ. Higher expression of neuroserpin inhibits the neuroprotective tPA and the generation of plasmin with subsequent reduction in the clearance of Aβ. These observations raise conflicting evidence on whether neuroserpin is neuroprotective or involved in AD progression. Thus, neuroserpin over-expression with subsequent reduction of tPA may propagate AD neuropathology.

**Graphical abstract:**

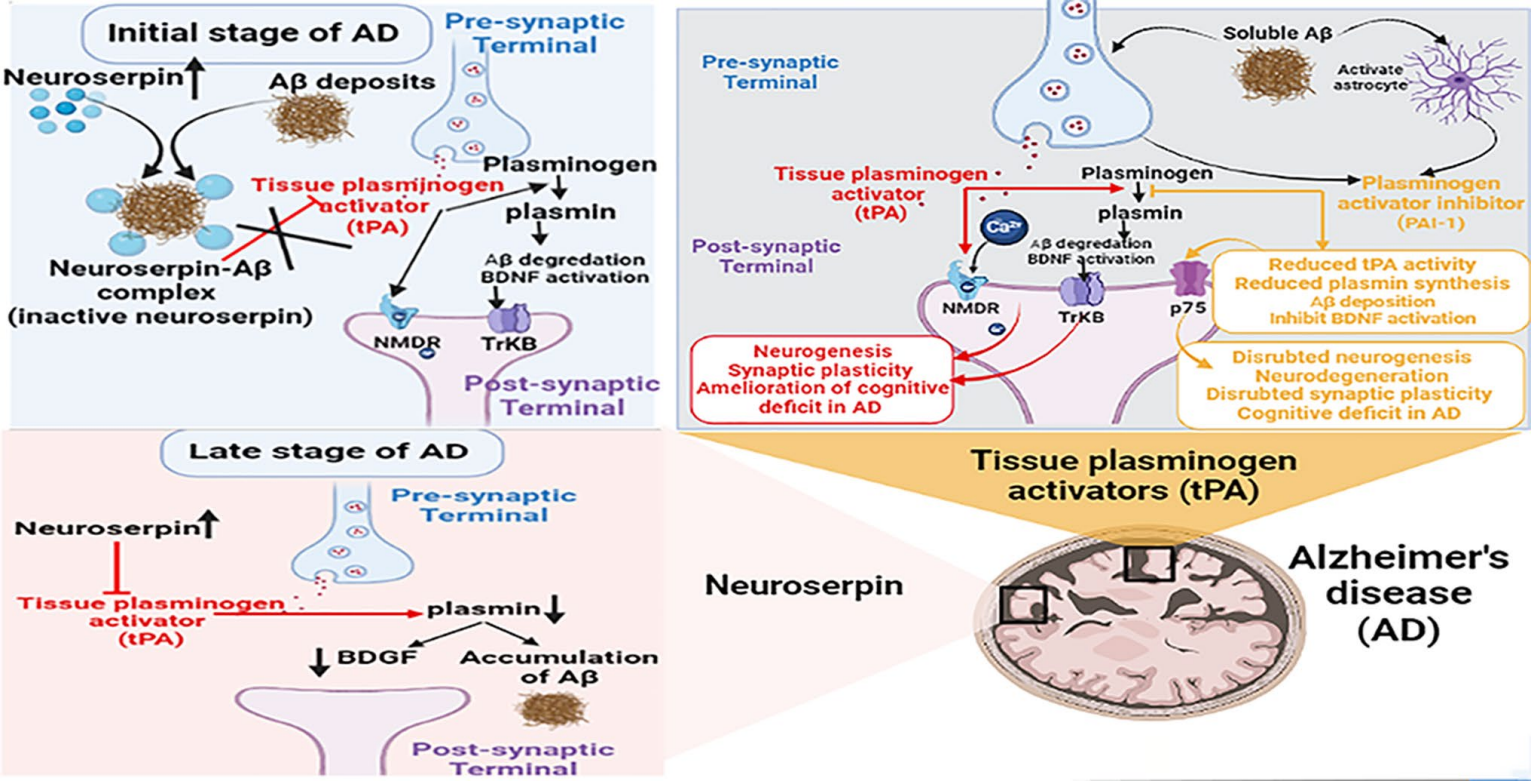

## Introduction

Alzheimer’s (AD) is a heterogeneous neurodegenerative disease with intricate neuropathological disorders. AD is the most common type of dementia, accounting for about 70% [1]. AD was first recognized by German psychiatrist Alois Alzheimer in 1906 in women with memory impairment [1]. AD affects 6% of the general population aged more than 65%, mainly in women, though 10% of early-onset dementia affecting people aged 30–60 years is attributed to AD [2]. Notoriously, AD affects 50 million people globally and is regarded as the 7th leading cause of death in the USA [2].

**Fig. 1:**
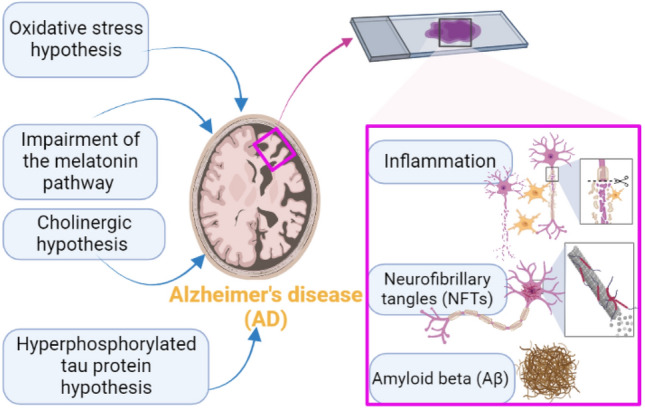
Pathophysiology of Alzheimer’s disease (AD)

AD is associated with extracellular deposition of amyloid beta (Aβ), mainly Aβ_1-42_ in the neocortex and hippocampus, leading to dementia and cognitive decline [3]. AD is characterized by intracellular accumulation of phosphorylated tau proteins as neurofibrillary tangles (NFTs) and extracellular deposition of Aβ as neuritic plaques [1, 4, 5]. These neuropathological features remain the chief criteria for AD [4]. However, different mechanisms are proposed for AD pathogenesis, including inflammation, oxidative stress, cholinergic dysfunction, and impairment of the melatonin pathway [4, 5] (Fig. 1).

These changes affect the lysosomal and endosomal clearance pathways by developing synaptic dysfunction and forming senile amyloid plaques derived from transmembrane amyloid precursor protein (APP) [6]. A defective neuronal clearance pathway due to the dysfunction of degradation enzymes could be a possible mechanism for accumulating Aβ and NFTs in AD [7]. Protease enzymes such as matrix metalloproteinase 9 (MMP-9), endothelin converting enzyme, neprilysin, insulin-degrading enzyme, and plasmin are involved in the degradation and clearance of Aβ and NFTs [8, 9]. Notably, plasmin formed from plasminogen by the action of tissue plasminogen activators (tPA) cleaves monomeric and fibrillar Aβ [10]. Besides, tPA is highly expressed in the brain and implicated in disorders including cerebellar motor learning, hippocampal long-term potentiation, amygdala-mediated anxiety, and hypothalamic endocrine dysfunction [11]. Significantly, tPA is inhibited by serine proteases, including neuroserpin, α2-antiplasmin, and protease nexin-1 [11]. In addition, tPA is highly expressed in brain areas with high plaque deposition, while plasmin level is reduced in AD [10, 11].

Plasminogen activator inhibitor-1 (PAI-1) regulates the expression of vascular tPA, which has a fibrinolytic effect [12]. It has been shown that tPA has pleiotropic properties in the central nervous system (CNS), including neurogenesis, synaptic plasticity, neurodegeneration, regulation of blood–brain barrier (BBB) permeability, and neurovascular coupling [13, 14]. Besides, neuroserpin is regarded as a potent inhibitor of tPA and implicated in the pathogenesis of AD through dysregulation of Aβ and NFTs clearance [13, 14].

Moreover, cyclin-dependent kinase 5 (cdk5) and its activator p35 are one of the chief tau phosphorylation systems involved in the regulation of neuronal polarity during the development of CNS [15]. Aβ promotes cdk5 activity, increasing intra-neuronal accumulation of tau protein [16]. The formation of the cdk5-p35 complex by Aβ triggers the neurodegeneration process [16]. A previous experimental study conducted by Alvarez et al. [17] demonstrated that inhibition of cdk5 attenuates Aβ-induced neuronal death.

Depending on this scientific rationale, the objective of the present narrative review was to explore the potential role of the tPA/neuroserpin axis in the pathogenesis of AD.

## Plasminogen activating system in AD

The plasminogen-activating system (PAS) is controlled by various enzymes, including tissue plasminogen activators (tPA) and urokinase plasminogen activator (uPA) for the synthesis of plasmin [18]. PAS is also regulated by plasminogen-activating inhibitor type 1 and type 2 (PA1-1) and (PAI-2), respectively [19]. Outside the CNS, PAS regulates fibrinolysis and homeostasis control [19]. PAS regulates AD pathogenesis; plasmin can cleave non-aggregated and aggregated fibrillar Aβ [20]. It has been reported that plasmin could attenuate Aβ-induced neuronal injury and death by enhancing Aβ clearance in the animal model study [21]. Jacobsen et al. [20] found that pharmacological inhibition of PAI-1 improves Aβ clearance via plasmin-mediated proteolysis in hippocampal slices from transgenic mice. Furthermore, plasmin activity is reduced in AD patients compared to healthy controls [11]. An in-vitro study observed that plasmin activity was reduced in the hippocampus of patients with AD due to a defect in the binding activity of plasmin. High neuronal membrane cholesterol impairs plasmin binding activity with the development of PAS dysfunction in AD [11]. Reduction of plasmin activity in AD patients is correlated with higher expression of ApoE, which is essential for neuronal cholesterol homeostasis and pathogenesis of AD [22]. Therefore, alteration of neuronal lipid raft through the expression of ApoE could be a possible mechanism in reducing plasmin activity and development of AD [21, 23]. A previous study by Ledesma et al. [24] confirmed that plasmin activity was reduced in the brains of AD patients. However, PAS and plasmin activity were not altered in AD patients’ temporal and frontal cortex homogenates [25]. A postmortem study involving 20 AD, 15 vascular dementia, and 20 healthy controls showed that plasminogen mRNA was normal in AD compared to controls [25]. This finding did not support the role of plasmin in the pathogenesis of AD. The cerebrospinal fluid (CSF) analysis study of AD patients and healthy controls revealed that tPA and PAI-1 levels were not altered compared to the controls [3]. The author concluded that plasmin activity in the CSF did not reflect the severity of AD pathology, so it was not valuable in diagnosing AD [3]. Notoriously, plasmin deficiency is not a secondary event but rather a primary event involved in the pathogenesis of AD [26].

### Role of tPA in AD

**Fig. 2:**
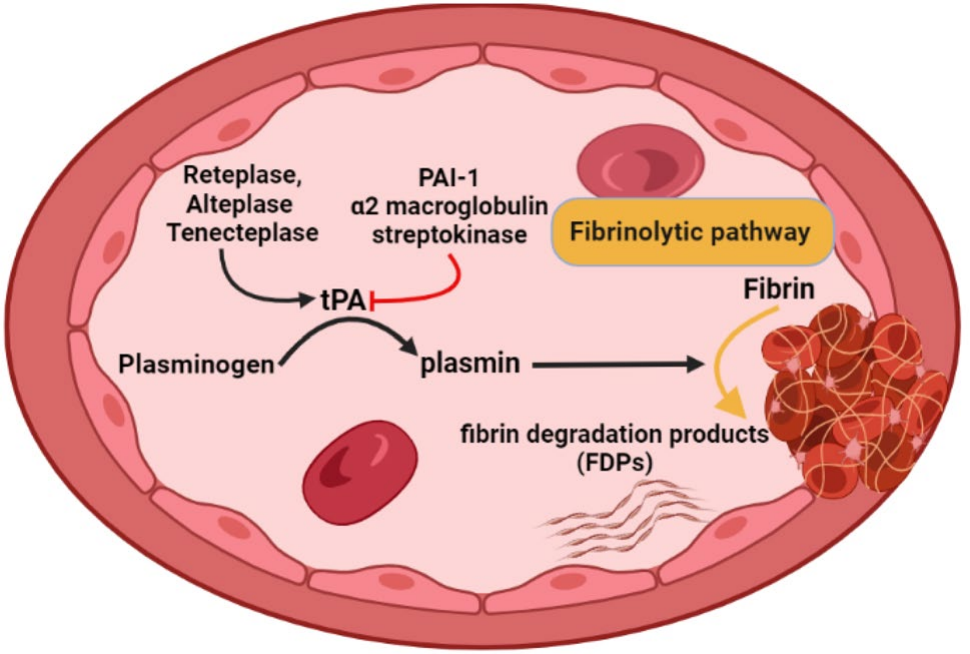
The physiological role of tissue plasminogen activators (tPA): tPA converts plasminogen to plasmin, leading to fibrin conversion to fibrin degradation products (FDPs). The tPA is modulated by plasminogen activator inhibitor-1 (PAI-1), α2 macroglobulin, and streptokinase

The action of tPA is modulated by PAI-1, α2 macroglobulin, and streptokinase (Fig. 2). tPA is categorized as a serine protease essential for clot lysis; thus, activation of tPA by thrombolytic agents such as reteplase, alteplase, and tenecteplase is integral in the management of acute ischemic stroke within 3–4 h, myocardial infarction, arterial thrombosis, pulmonary embolism, and deep vein thrombosis [27, 28].

Principally, tPA plays a different function in the CNS as neurons and astrocytes express it. It regulates synaptic growth, neuronal migration, synaptic plasticity, modulation of neurotransmission, and cognitive enhancing effects [29]. Remarkably, tPA is synthesized, stored in the neurons, and released upon neuronal depolarization [30]. Neuronal tPA enhances the conversion of plasminogen to plasmin; this function is controlled by inhibitors of PAS, including PA1-1 and neuroserpin [31]. Experimental evidence confirmed that tPA and uPA exert neuroprotective effects independent of plasmin generation after ischemic stroke [31]. Rapid release of tPA from presynaptic neurons following brain ischemic events protects the synapses from the harmful effects of ischemia. In addition, released uPA during recovery promotes neuronal and synapse repairs [31]. These findings suggest neuroprotective and neuro-restorative effects of tPA and uPA. Thus, PAS could be a potential target in the modulation of pathologic processes in various neurodegenerative diseases.

Moreover, the plasminogen level in the AD brain was documented to be not altered compared to the matched controls due to impairment of plasminogen activation by tPA [32]. The underlying cause for the reduction of tPA is due to the co-localization of tPA with Aβ, which impairs tPA activity [32]. However, the tPA level did not change, but its activity was dramatically reduced in the AD brain compared to the controls [32]. TPA activity is notably reduced with aging, which may provoke AD development. TPA activity is significantly reduced in the AD brain compared to the control [33].

### Role of PAI-1 in AD

It has been observed that PAI-1 activity and level are increased in AD according to the findings from animal and human studies [33, 34]. A case–control study illustrated that higher expression of PAI-1 and low tPA/PAI-1 ratio was observed in diabetic patients with cognitive impairment and memory dysfunction [34]. A higher PAI-1 plasma level is regarded as a potential biomarker in the detection of AD [35]. PAI-1 plasma level is negatively correlated with cognitive function [35]. A case–control study confirmed that AD patients' PAI-1 plasma level was reduced compared to controls [35]. In addition, the PAI-1 level was not changed in the frontal cortex in mice and human AD brains [36]. Higher PAI-1 and low tPA levels correlate with Aβ deposition in AD. Hence, reduced tPA may be the causal mechanism in AD development. Supporting this notion, tPA-deficient mice had defects in the removal of injected human Aβ_1-42_ as compared with wild-type mice [36].

Similarly, a more significant accumulation of Aβ_1-42_ was demonstrated in mice with genetically reduced endogenous tPA production [37]. It has been shown that tPA activity and expression are higher around Aβ plaques in the brains of human APP over-expressing Tg2576 mice, which have undergone a genetic ablation of tPA [37]. Reduction of tPA in the brain triggers the accumulation of Aβ_1-42_ with significant expression of a synaptic function-associated protein involved in developing synaptic dysfunction and cognitive deficits [37]. Different evidence from preclinical studies proposed that failure of synaptic function may occur earlier before the progression of neuronal loss and development of AD [38]. A cohort study illustrated that miR-210-3p, which reflects synaptic dysfunction, increased in AD patients [38]. Tian et al. [39] observed that blood extracellular vesicles carrying synaptic function-associated protein are potential biomarkers for diagnosing AD. A cross-sectional study showed that extracellular vesicle plasma levels were reduced in AD patients [40]. These findings suggest that synaptic dysfunctions precede symptomatic AD, and biomarkers of synaptic dysfunctions could be helpful in the detection of high-risk subjects.

### The interaction between Aβ and PAS

It has been suggested that plaques play a critical role in sequestering the soluble form of Aβ to reduce its neurotoxic effect in AD [41]. Later on, plaque sequestration capacity is reduced with the time of AD progression, and soluble Aβ can diffuse extracellularly, causing extensive synaptic dysfunction and neuronal injury [42]. Sciaccaluga et al. [43] suggested that Aβ oligomers trigger intracellular and extracellular neurotoxicities through interaction with cell membrane ion channels and receptors. These pathological changes promote a profound imbalance between inhibitory and excitatory neurotransmitters with hyper-excitability development in AD [43]. In this state, hyper-excitability contributes to the deposition of Aβ and the development of neurodegeneration. Thus, an imbalance of neurotransmissions is attributed to oligomer Aβ deposition, and the reverse is invalid [40]. An imaging study and computational neuronal modeling involving AD patients demonstrated significant subpopulation alteration in the excitatory/inhibitory axis concerning the severity of Aβ deposition compared to the controls [40]. Therefore, synaptic dysfunction with the development of excitatory/inhibitory imbalance promotes Aβ-induced neuronal injury in AD.

Moreover, apolipoprotein E (ApoE) predisposes to the development and progression of AD in about 40% and is involved in the impairment of Aβ clearance [44, 45]. Soluble Aβ density isolated from AD patients is correlated with synaptic dysfunction and cognitive deficits. Soluble Aβ effect on tau protein phosphorylation and neurotoxicity had been confirmed in crossing human APP with tau transgenic mice [44]. Therefore, monoclonal antibodies against soluble Aβ could be effective against the progression of AD pathogenesis [46]. Human clinical trials that tested monoclonal antibodies, including solanezumab and bapineuzumab, against soluble Aβ were evaluated in managing mild-moderate AD [46]. Solanezumab in the Phase III trial was effective in treating AD patients. In addition, monoclonal antibodies, including gantenerumab, which binds fibrillary, and crenezumab, which binds soluble Aβ, revealed promising effects in preventing AD in pre-symptomatic susceptible subjects [46]. Aducanumab was the monoclonal antibody approved by the FDA in 2003 to manage mild AD [47].

**Fig. 3:**
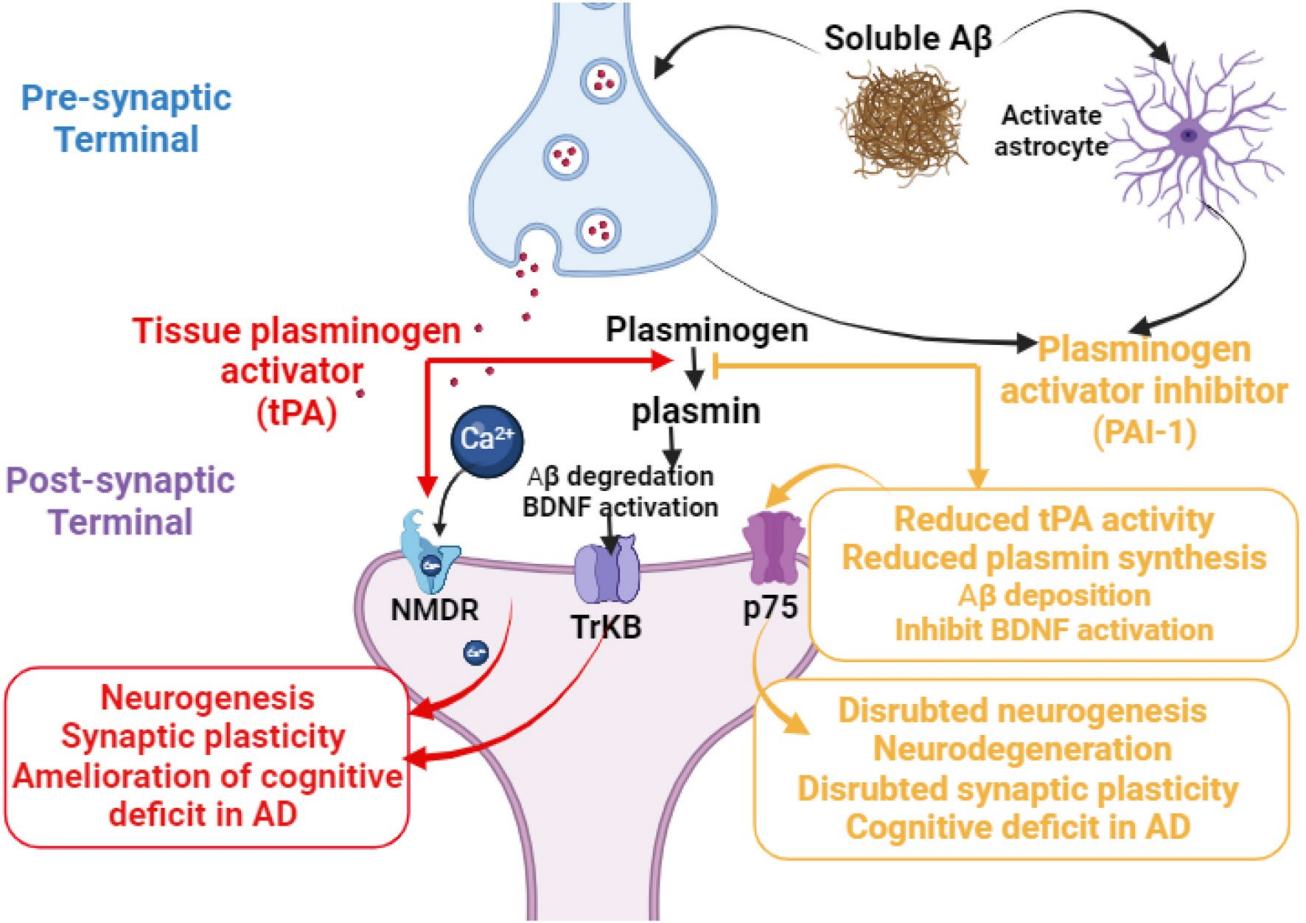
Role of tissue plasminogen activators (tPA) in Alzheimer’s disease: neuronal tPA enhances the conversion of plasminogen to plasmin that prevents Aβ deposition and enhances the activation of neuroprotective brain-derived neurotrophic factor (BDNF). Activation of transient receptor kinase B (TrKB) by BDNF and N-methyl-D-aspartate (NMDR) by tPA promotes neurogenesis and synaptic plasticity with amelioration of cognitive deficit in AD-soluble Aβ activates plasminogen activator inhibitor (PAI-1) that responsible for the accumulation of Aβ and inhibition activation of BDNF leading to activation of p75 that disrupt synaptic plasticity with the development of neurodegeneration and cognitive deficits

It has been shown that soluble Aβ in AD affects the protective role of tPA through activation of PAI-1, which is involved in the accumulation of Aβ and inhibition activation of neuroprotective brain-derived neurotrophic factor (BDNF) [48]. Pro-BDNF is cleaved and activated to BDNF by plasmin that regulates the memory process and neuronal activity. Thus, through activation of the BDNF pathway, tPA improves synaptic plasticity in AD [49]. Therefore, modulation of tPA/PAI-1 could be a therapeutic strategy in managing AD (Fig. 3). These pathological changes through activation of p75NTR disrupt long-term potentiation and synaptic plasticity with the development of neurodegeneration and cognitive deficits. As well, activation of transient receptor kinase B (TrKB) by BDNF and N-methyl-D-aspartate (NMDR) by tPA promote neurogenesis and synaptic plasticity with amelioration of cognitive deficit in AD [50, 51].

**Fig. 4:**
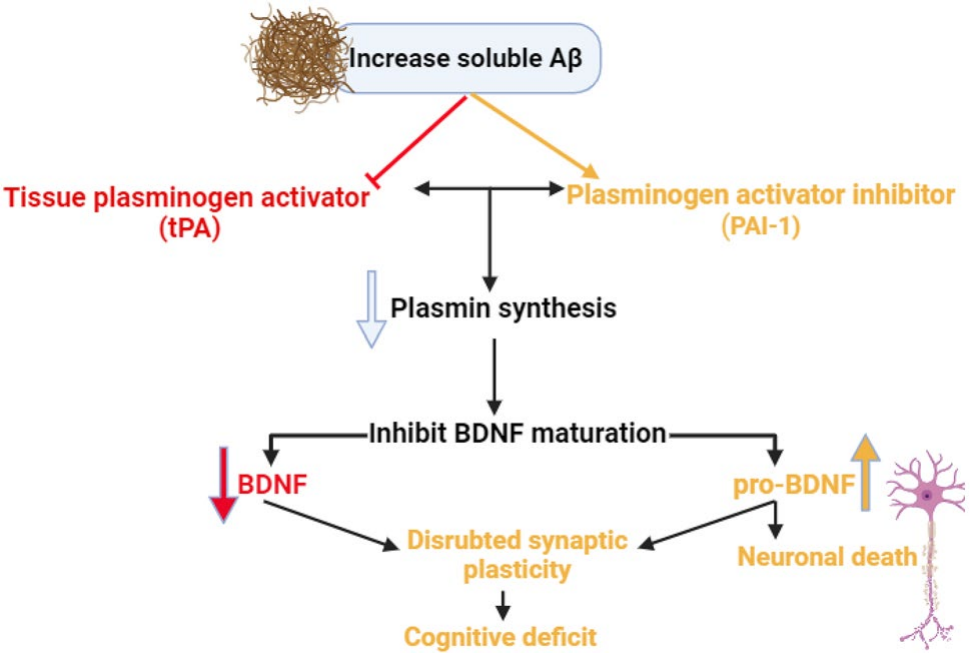
Effects of soluble amyloid beta (Aβ) on the plasminogen pathway in Alzheimer’s disease (AD). Brain-derived neurotrophic factor (BDNF)

Indeed, soluble Aβ is more toxic than non-soluble ones in the neurons, leading to cognitive dysfunction through induction of synaptic dysfunction, disruption of signaling pathways, and AD neuropathology [41, 52]. It has been shown that soluble Aβ affects synaptic dysfunction through inhibition of tPA and activation of PAI-1 with subsequent effect on the expression of BDNF (Gregnani et al. 2020). An experimental study confirmed that soluble Aβ impairs the expression of BDNF through modulation of the tPA/PAI-1 axis in the AD mouse model [53]. Thus, the exaggeration of PAI-1 activity in AD attenuates the expression of tPA and plasmin generation from plasminogen [53]. Further reduction of BDNF due to the reduction of plasmin leads to neuronal atrophy and neuronal deaths [54] (Fig. 4).

It has been reported that a progressive increase of Aβ level during AD neuropathology is correlated with the over-production of PAI-1 with subsequent reduction of plasmin and tPA levels and activities [55]. In turn, the reduction of plasmin and tPA activities promotes Aβ accumulation by reducing Aβ clearance [56]. As well, Aβ-induced tPA dysfunction leads to neurovascular disorders [57]. Thus, exogenous tPA or pharmacological inhibition of PAI-1 can attenuate Aβ-induced neurovascular dysfunction [56]. Likewise, Aβ activates astrocyte expression of PAI-1 in the cerebral cortex [58]. Injection of exogenous Aβ in mice brains promotes the expression of PAI-1 [58]. Besides, different studies revealed that pro-inflammatory cytokines reduce tPA and increase the formation of PAI-1 [59, 60]. For example, tranexamic acid has an anti-inflammatory effect by inhibiting tPA by reducing plasmin-mediated complement activation. Since tranexamic acid does not affect uPA, it increases uPA-mediated plasmin generation and complements activation by releasing pro-inflammatory cytokines [61]. Besides, IL-6 activates the release of PAI-1 and promotes the development of endothelial dysfunction during cytokine-releasing syndrome [60]. Notably, higher expression of pro-inflammatory cytokines and inflammatory processes around Aβ plaques promote the expression of PAI-1 in the brain of AD patients [61]. Thus, anti-inflammatory agents might be a therapeutic strategy to prevent Aβ accumulation.

Taken together, inherited or acquired defect in the brain plasmin/tPA pathway promotes the accumulation of Aβ, which stimulates the expression and generation of PAI-1. In this state, activated PAI-1 reduces the neuroprotective effect of plasmin with further Aβ accumulation. Therefore, there is a positive feedback loop between PAI-1 expression and the pathogenesis of AD.

## Neuroserpin in AD

Neuroserpin is a protease inhibitor involved in various physiological functions, including synapse formation and axonal growth [62]. Neuroserpin is mainly expressed in the CNS, responsible for synaptogenesis and axonogenesis during embryonic life and regulation of synaptic plasticity in adults [62]. Neuroserpin is also expressed outside the CNS in the pancreas, testis, liver, kidney, and immune cells [63]. Outside the CNS, neuroserpin regulates the expression of chemokines by T cells, migration of immune cells, T cell activation, and regulation of immunoinflammatory response [62]. It has been reported that neuroserpin is intricate in the pathogenesis of atherosclerosis and vascular inflammation [64, 65]. Remarkably, neuroserpin has potent anti-inflammatory effects through modulation of T cell activation and invasion during vascular inflammation and formation of atherosclerotic plaques [64, 65]. A cross-sectional study revealed that neuroserpin level was higher in patients with rheumatoid arthritis and correlated with disease severity [65]. Therefore, increasing neuroserpin levels during systemic inflammatory disorders could be a compensatory mechanism to mitigate inflammatory reactions through modulation of T cell response [64]. Loef et al. [66] observed that neuroserpin had an anti-inflammatory effect by inhibiting T cell proliferation and clustering. This effect is mediated by inhibiting the expression of tPA and plasmin-mediated release of annexin-A and F-actin, which are involved in T cell proliferation [66].

These observations illustrated that peripheral neuroserpin has systemic anti-inflammatory effects and regulates immunoinflammatory response.

In the CNS, neuroserpin expression is increased progressively at the perinatal period. It is sustained in adulthood in all brain regions, mainly the hippocampus, neocortex, olfactory bulb, and amygdala, which engage with memory and learning [67]. Neuroserpin plays a crucial role in the development and maturation of CNS, though its function in the mature brain is linked with synaptogenesis and synaptic plasticity [67]. An experimental study demonstrated that neuroserpin-knockout mice experience cognitive dysfunction due to impairment of synaptic function and hippocampal dysfunction [68].

Markedly, neuroserpin polymorphism and dysfunction trigger its intracellular accumulation with the development of wide-spectrum diseases like familial encephalopathy with neuroserpin inclusion body (FENIB) [69]. Alterations in the expression and activity of neuroserpins are associated with the development of different neuropathological disorders. For example, neuroserpin deficiency increases neuronal injury and infarct size in mice with experimental ischemic stroke [70]. It has been shown that administration of neuroserpin in the experimental animal with an ischemic stroke leads to a neuroprotective effect, as evidenced by the reduction of cerebral infarct size [71]. Indeed, higher neuroserpin expression correlates with better clinical outcomes in patients with ischemic stroke [72]. Besides, neuroserpin has a neuroprotective effect against NMDA-induced neurotoxicity [73]. Likewise, neuroinflammatory response and reaction are reversed by neuroserpin, which maintains BBB integrity [74]. The neuroprotective role of neuroserpin is related to the inhibition of tPA-induced neuroinflammation, microglial activation, and BBB injury [72]. Moreover, neuroserpin is implicated in the pathogenesis of bipolar disorders. A case–control study illustrated that neuroserpin plasma levels were lower in patients with bipolar disorders compared to healthy controls [75].

**Fig. 5:**
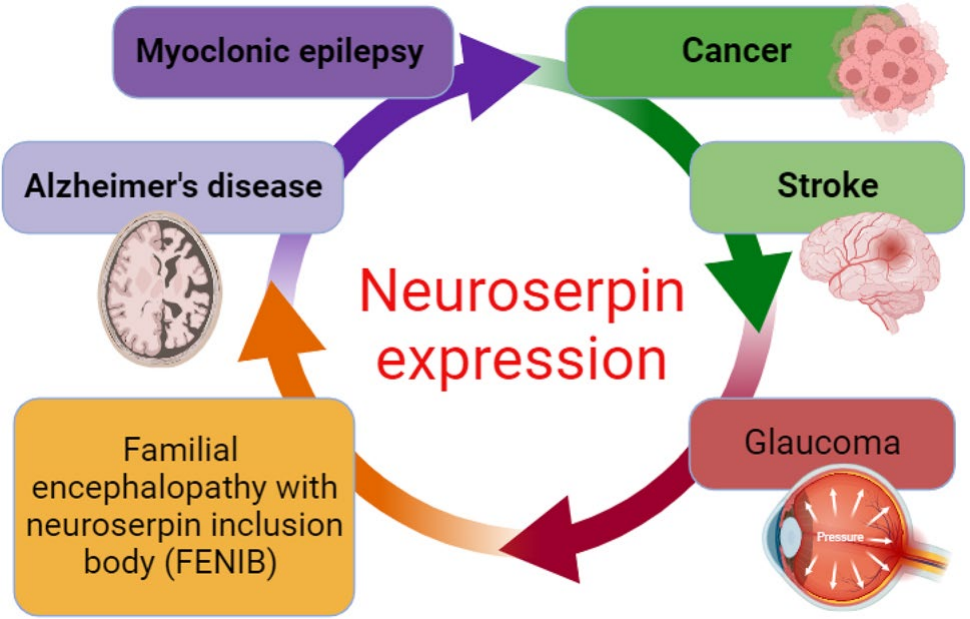
Role of neuroserpin in the development of various diseases

Genetic variations of neuroserpin expression are linked with the development of various neurological disorders, including FENIB, myoclonic epilepsy, AD, cancer, and glaucoma (Fig. 5) [69].

**Fig. 6:**
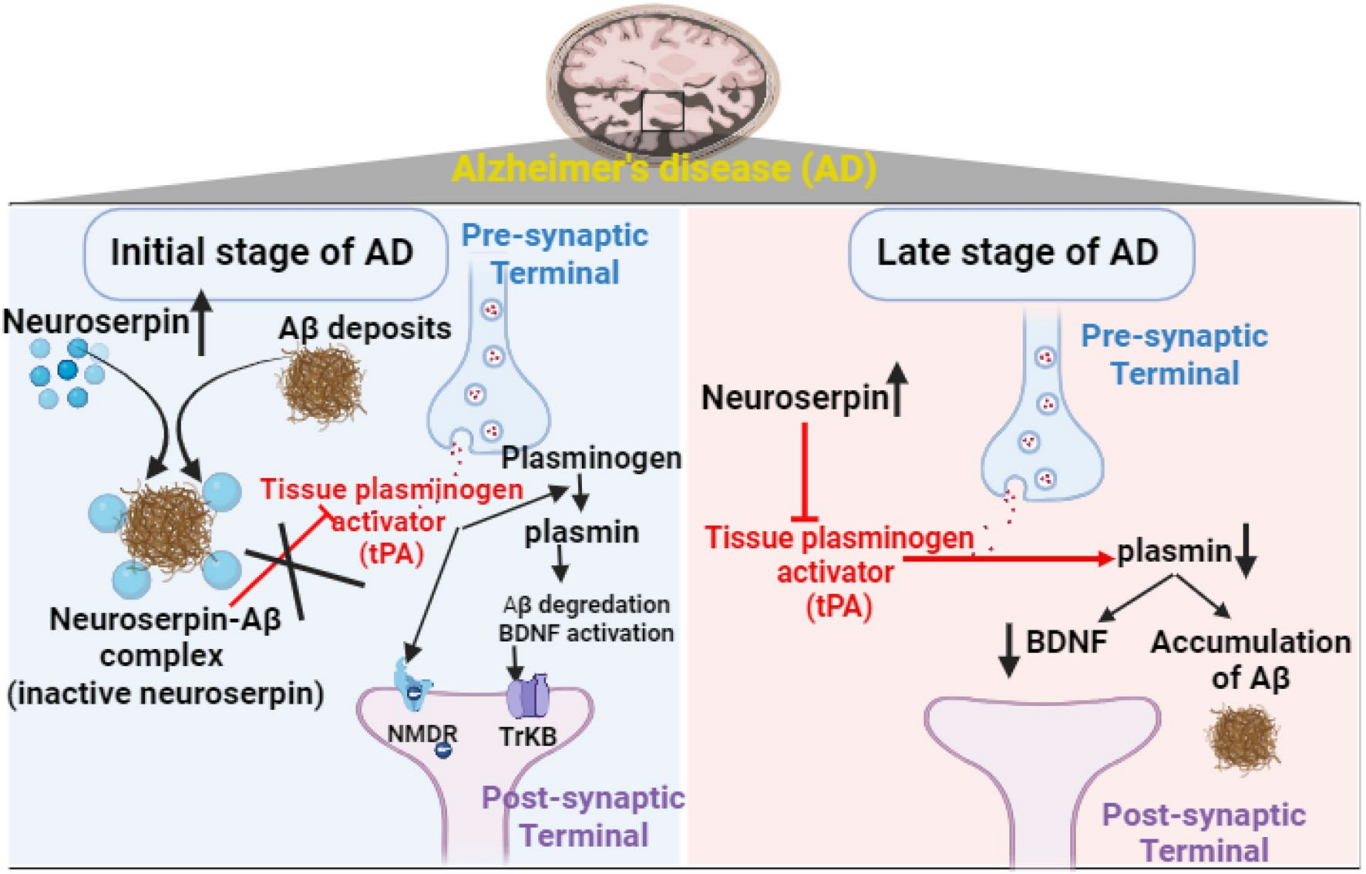
Role of neuroserpin in the pathogenesis of Alzheimer’s disease: Neuroserpin forms a binary complex with amyloid beta (Aβ)1–42, which inactivates neuroserpin as an inhibitor of tissue plasminogen activators (tPA) that protect the neurons in AD. Increasing neuroserpin levels is linked with the reduction of brain-derived neurotrophic factor (BDNF) in the late stage of AD due to the inhibition of plasmin

Neuroserpin plays a critical role in the pathogenesis of AD; it regulates the expression and accumulation of Aβ [76]. Neuroserpin forms a binary complex with Aβ_1-42,_ which inactivates neuroserpin as an inhibitor of tPA and blocks the polymerization process [77]. However, neuroserpin has a role in accelerating the accumulation of Aβ, which is dissimilar from that of mature amyloid fibril. In vitro studies demonstrated that neuroserpin attenuates Aβ-induced neurotoxicity [77]. Therefore, the interaction between neuroserpin and Aβ_1-42_ induces the formation of the non-toxic oligomer to protect the neurons in AD. An experimental study conducted by Fabbro et al. [78] illustrated that neuroserpin-deficient mice have a higher reduction in the accumulation of Aβ_1-42,_ suggesting a possible role of neuroserpin in the aggregation of Aβ and the development of AD. Higher expression of neuroserpin inhibits the neuroprotective tPA and generation of plasmin with the reduction in the clearance of Aβ (Fig. 6) [78]. Ablation of neuroserpin in mice improves clearance of Aβ and reduces Aβ accumulation through activation of tPA [78]. Mutation and conformational changes in neuroserpin are associated with the onset and severity of dementia in patients with neurodegenerative diseases [79].

Moreover, it has been suggested that Aβ acts as an enhancer for the polymerization of neuroserpin [80]. Increasing neuroserpin level is also associated with neuronal toxicity and uncontrolled excitation [81]. These observations raise conflicting evidence of whether neuroserpin is neuroprotective or involved in AD progression. Previous studies implicate neuroserpin’s role in AD pathogenesis [82]. A case–control study demonstrated that neuroserpin level in CSF was higher in AD patients compared to controls [82]. Thus, CSF neuroserpin level could be a diagnostic biomarker for AD. CSF neuroserpin level is also correlated with tau protein phosphorylation biomarkers in the CSF [82].

Similarly, CSF neuroserpin and plasminogen levels are increased in patients with AD and mild cognitive impairment but without significant differences [83]. Notably, the expression of neuroserpin is reduced in the advanced stage of AD after extensive neuronal loss. Distribution of inhibitors and activators of tPA were assessed in brain tissues from 20 AD patients, and 20 healthy controls showed that neuroserpin expression was reduced, while PAI-1 was increased in temporal and frontal cortices [84]. Higher expression of neuroserpin is associated with a significant reduction of tPA activity with subsequent Aβ accumulation [78, 84]. It has been observed that tPA/plasmin activity was decreased in AD patients compared to controls [78, 84].

Moreover, dysregulation of autophagy and the development of endoplasmic reticulum stress are associated with the accumulation of misfolded proteins, which affect neuroserpin activity in AD [85, 86]. Remarkably, thyroid hormone induces the expression of neuroserpin in AD [85]. Analysis of brain tissues of AD patients in a postmortem study revealed that the expression of thyroid hormone receptors was higher than controls [87]. Bavarsad et al. [87] suggested that thyroid disorders, either hyperthyroidism or hypothyroidism, may be involved in the pathogenesis of AD as thyroid hormones affect memory and cognitive functions [88, 89]. A prospective cross-sectional study of 69 patients showed that thyroid-stimulating hormone levels and thyroid hormones correlated with cerebral tau burden and cognitive dysfunction [88]. These findings implicate thyroid hormones in the pathogenesis of AD. The underlying cause for the association between thyroid disorders and AD is the upregulation of neuroserpin expression and AD neuropathology. Moreover, increasing neuroserpin levels is linked with the reduction of BDNF in AD due to the inhibition of plasmin, which is involved in the activation conversion of pro-BDNF to BDNF [90].

These observations proposed that increased neuroserpin expression in the initial stage of AD might be protective against aggregation and accumulation of Aβ. However, increased neuroserpin expression in the late stage of AD may increase disease severity by inhibiting tPA/plasmin activity [77].

## The link between neuroserpin and tPA/plasmin in AD

The potential link between neuroserpin and tPA/plasmin in AD has been reported in different studies [91]. Neuroserpin, via inhibition of tPA and, to a lesser extent, uPA induces accumulation of Aβ [91]. tPA participates in various physiological processes regardless of plasminogen activation, including synaptic plasticity, neuronal growth, and brain development [92]. However, tPA is also intricate in many neuropathological disorders; it participates in the development of excitotoxicity and associated neuronal injury following ischemic stroke [93–95]. The physiological role of neuronal tPA is protective, whereas pathological induction release of tPA is associated with detrimental neurological disorders [91]. Therefore, modulation of neuronal tPA by neuroserpin is essential to find the effect of tPA on synaptic plasticity. Neuroserpin attenuates tPA-induced cell proliferation and migration in vitro and visual cortical plasticity [91]. However, in animal model studies, tPA activity was documented to be unaffected by neuroserpin in mice [96].

Nevertheless, neuroserpin is neuroprotective against tPA-mediated neuronal injury during cerebral ischemic events [22]. Neuroserpin is critical in regulating synaptic connection, neuronal differentiation, synaptogenesis, and brain development, mainly in the hippocampus [22, 97]. However, over-expression of neuroserpin is associated with neuronal maturation dysfunction [98]. Indeed, higher expression of neuroserpin in AD pathogenesis had been documented in a previous study [78], though the underlying cause for the elevation of neuroserpin in AD was not fully elucidated. Up-regulation of neuroserpin during Aβ accumulation could be a compensatory mechanism to reduce Aβ-induced neurotoxicity [80, 97]. Alterations of neuronal cholesterol by the long-term effect of statins may induce neuroserpin aggregation and dysfunction [99]. As well, thyroid disorders, ischemic disorders, and neurodegenerative processes affect the expression of neuroserpin [22, 85, 87]. Thus, neuroserpin over-expression with subsequent reduction of tPA may propagate AD neuropathology.

In this bargain, neuroserpin over-expression and tPA dysfunction are not merely the sole mechanism in AD pathogenesis. Searching for the underlying causes of neuroserpin and tPA dysregulation could open a new window regarding the pathogenesis and management of AD.

## Conclusions

AD is the most common type of dementia characterized by intracellular accumulation of phosphorylated tau proteins and deposition of Aβ. A defective neuronal clearance pathway due to the dysfunction of degradation enzymes could be a possible mechanism for the accumulation of Aβ. Plasmin formed from plasminogen by the action of tissue plasminogen activators (tPA) cleaves Aβ. tPA is highly expressed in brain areas with high plaque deposition that is reduced in AD. tPA is inhibited by serine proteases, including neuroserpin. Neuronal tPA enhances the conversion of plasminogen to plasmin; this function is controlled by inhibitors neuroserpin. Plasmin activity is reduced in AD patients and is correlated with higher expression of ApoE, which is essential for the pathogenesis of AD. The underlying cause for the reduction of tPA is due to the co-localization of tPA with Aβ, which impairs tPA activity. PAI-1 activity is increased in AD due to an increase in Aβ level, which is correlated with the over-production of PAI-1. Reducing plasmin and tPA activities promote Aβ by reducing Aβ clearance. Thus, there is a positive feedback loop between PAI-1 expression and the pathogenesis of AD.

Neuroserpin plays a critical role in the pathogenesis of AD; it acts as an inhibitor of tPA. Higher expression of neuroserpin inhibits the neuroprotective tPA and generation of plasmin with a reduction in the clearance of Aβ. The underlying cause for the elevation of neuroserpin in AD was not fully elucidated and may be a compensatory mechanism to reduce Aβ-induced neurotoxicity. Thus, neuroserpin over-expression with subsequent reduction of tPA may propagate AD neuropathology. In this bargain, neuroserpin over-expression and tPA dysfunction are not merely the sole mechanism in AD pathogenesis. Findings from this review cannot give a conclusion; thus, experimental, preclinical, and prospective studies are recommended in this regard.

## Data Availability

Not applicable.
